# The role of superoxide anions in the development of distant tumour recurrence

**DOI:** 10.1038/sj.bjc.6603436

**Published:** 2006-11-07

**Authors:** M ten Kate, J B C van der Wal, W Sluiter, L J Hofland, J Jeekel, P Sonneveld, C H J van Eijck

**Affiliations:** 1 Department of Surgery, Erasmus MC, Dr Molewaterplein 40, 3015 GD Rotterdam, The Netherlands; 2 Department of Biochemistry, Erasmus MC, Rotterdam, The Netherlands; 3 Department of Internal Medicine, Erasmus MC, Rotterdam, The Netherlands; 4 Department of Hematology, Erasmus MC, Rotterdam, The Netherlands

**Keywords:** surgical trauma, ROS, tumour cell, microvascular endothelium, adhesion

## Abstract

We hypothesise that reactive oxygen species (ROS) released from activated polymorphonuclear leucocytes during surgery play a crucial role in enhanced tumour recurrence seen after surgery. Therefore, the effect of ROS on adhesion of tumour cells to microvascular endothelium in a reproducible human *in vitro* model was studied. Preincubation of microvascular endothelial cells with the superoxide anion producing xanthine–xanthine oxidase complex significantly increased adhesion of the human colon carcinoma cells HT29 (167% *vs* control, *P*<0.01), Caco2 (164% *vs* control, *P*<0.01) and of the pancreas carcinoma cells PanC1 (180% *vs* control, *P*<0.01). Addition of the antioxidant enzymes superoxide dismutase or catalase significantly decreased tumour cell adhesion (*P*<0.01). Exposure of endothelial cells to superoxide anions increased the apoptotic rate to 7.9 times the normal rate. Additionally, exposure increased expression of the endothelial adhesion molecules E-Selectin, ICAM-1, and VCAM-1 of maximally 170% *vs* control (*P*<0.01). In conclusion, this study shows that superoxide anions promote the adherence of tumour cells to the microvasculature by inducing endothelial apoptosis that subsequently induces the expression of various adhesion molecules for tumour cells. This indicates that by tackling the production of ROS preventing tumour recurrence at distant sites might be feasible.

Despite the introduction of new treatment modalities for gastrointestinal malignancies during the last decades surgery remains the principal therapy for most gastrointestinal malignancies, although the recurrence rates after intentionally curative surgery are high (August *et al*, 1984; Midgley and Kerr, 1999; Wayne *et al*, 2002; Link *et al*, 2005).

Operative trauma in itself may favour development of tumour recurrence. This relation between abdominal surgery and locoregional tumour recurrence was investigated in previous *in vivo* and *in vitro* experiments. These studies illustrated that surgical trauma enhanced locoregional tumour recurrence and that this phenomenon involved a dose–response relation, that is, severe trauma was associated with a higher locoregional tumour recurrence rate compared to mild trauma (Busch *et al*, 1993; Bouvy *et al*, 1997; van den Tol *et al*, 1998; van Rossen *et al*, 1999, 2000). Further experiments demonstrated that abdominal surgical trauma provoked a local inflammatory reaction with influx of mainly polymorphonuclear cells (PMN). These activated PMN produced reactive oxygen species (ROS) which are found to play an important role in the observed enhanced locoregional tumour recurrence in which binding of the tumour cells to the mesothelium is an essential step (van Rossen *et al*, 1999, 2000).

The inflammatory reaction caused by abdominal surgical trauma is not confined to the abdominal cavity, but spreads out systemically (Weese *et al*, 1986; Baigrie *et al*, 1992; Yokota *et al*, 1995; Aosasa *et al*, 2000; Shiromizu *et al*, 2000; Pross *et al*, 2002; Ure *et al*, 2002). So is it found that during and shortly after major surgery, the peripheral blood level of elastase, which is an indicator of PMN activity, is elevated (Oka *et al*, 1994; Noshima *et al*, 1997; Varga *et al*, 1997; Hildebrandt *et al*, 2003; Miyaoka *et al*, 2005). Furthermore, major abdominal surgery results in an elevated PMN concentration at distant sites, for example in the lung leading to a distant inflammatory reaction (Fosse *et al*, 1987). Therefore, surgical trauma may not only promote local tumour recurrence, but also tumour recurrence at distant sites.

Cancer dissemination is frequently accomplished via the blood stream. Although many circulating tumour cells fail to survive this phase of the metastatic cascade, the establishment of metastases depends upon the arrest of surviving cells and their exit from the circulation, which involves adhering to and crossing the barriers imposed by the microvascular endothelium and extracellular matrix (Weiss, 1985; Fosse *et al*, 1987).

Based on the previous studies that demonstrate an important role for ROS in locoregional tumour recurrence after surgical trauma, combined with the systemic inflammatory process after surgical trauma, we hypothesise that ROS enhance distant tumour recurrence by increased tumour cell adhesion to the endothelium.

In this study therefore, we investigate the influence of ROS on tumour cell–endothelial cell interactions. The underlying mechanism of the enhanced adhesion by PMN-derived ROS will be further elucidated. Two tumour cell types were used, namely colon and pancreas carcinoma cells to assess the effect of superoxide anions on tumour cell–endothelial cell interactions with focus on the expression of a variety of cellular adhesion molecules and the occurrence of apoptosis of both the tumour and microvascular endothelial cells (MEC).

## MATERIALS AND METHODS

### Cells

Human MECs of the lung were purchased from Cambrex (Verviers, Belgium) at passage 4 and maintained in EGM-2-MV Bullet kit according to the manufacturer's instructions at 37°C, 95% relative humidity and 5% CO_2_. Confluent monolayers were passaged by 0.025% trypsin/0.01% ethylenediaminetetraacetic acid (EDTA) and cells were used up to passage 8.

The human colon carcinoma cell lines HT29 and Caco2 and the human pancreas carcinoma cell line PanC1 were grown in EGM-2-MV Bullet kit as well in order to create similar conditions and maintained by serial passage after trypsinisation using 0.05% trypsin/0.02% EDTA (Gibco, Breda, The Netherlands).

Before the adhesion assay, tumour cells were trypsinised and maintained in suspension for 2 h to regenerate cell-surface proteins.

### Reactive oxygen species and scavengers

In this study, the xanthine (X)–xanthine oxidase (XO) complex was used in a concentration of 100 *μ*M and 30 mU ml^−1^, respectively (Sigma-Aldrich, Zwijndrecht, the Netherlands), to produce superoxide anions.

Superoxide anions were inactivated by the addition of 400 U ml^−1^ superoxide dismutase (SOD) (Roche Applied Science, Almere, The Netherlands) that converts superoxide anions into molecular oxygen and hydrogen peroxide. As hydrogen peroxide may itself affect tumour cell adhesion, 400 U ml^−1^ catalase (Sigma-Aldrich, Zwijndrecht, The Netherlands) was added to the *in vitro* model alone or in combination with SOD to decompose any hydrogen peroxide.

### Ferricytochrome *c* reduction assay

To assess production of superoxide anions generated by the combination of X and XO in our model we used the ferricytochrome *c* reduction assay (Markert *et al*, 1984). This assay was performed in phenol red-free and phosphate-buffered Hank's Balanced Salt Solution (Invitrogen, Breda, the Netherlands) with 5% foetal calf serum, as phenol red and pH changes effect the assay. After addition of 75 *μ*M cytochrome *c* (Roche Applied Science, Almere, The Netherlands) the change in absorbance at 550 and 540 nm (reference) was continuously recorded by the thermostatted Versamax microplate reader (Molecular Devices) for 125 min at 37°C.

### Adhesion assay

To quantify tumour cell adhesion to MEC, a standardised cell adhesion assay was developed as described before (Catterall *et al*, 1994). Briefly, endothelial monolayers were established in 96-well microtitre plates (Perkin Elmer, Groningen, The Netherlands). To do this, confluent cells were trypsinised and 2 × 10^4^ endothelial cells were added to each well.

The plates were incubated at 37°C, 95% relative humidity, 5% CO_2_, and medium was daily replaced by fresh medium. Microvascular endothelial cells reached confluence in 3–4 days as determined by light microscopy.

To determine the effect of ROS on tumour cell adhesion, endothelial monolayers were preincubated with varying doses of X and XO, during varying times. Untreated monolayers served as controls. Tumour cells were preincubated or not with the X–XO complex for 12 h before the adhesion assay.

Appropriate SOD and/or catalase were added to the model system to assess ROS specificity of the effects.

To quantify tumour cell adhesion, tumour cells (1 × 10^6^ cells ml^−1^) were labelled with calcein-AM (Molecular Probes, Leiden, The Netherlands) and 3 × 10^4^ cells per well were added. Plates were centrifuged for 1 min at 80 × *g* and incubated at 37°C for 1 h. After this, the wells were washed twice with medium. The remaining fluorescence per well was measured on a Perkin Elmer plate reader using a wavelength of 485 nm for excitation and 530 nm for emission respectively.

### Enzyme immunoassay (EIA)

Endothelial and tumour cells were grown to confluence as described for the adhesion assays in 96-well flat-bottomed microtiter plates (Becton & Dickinson, Erembodegem, Belgium). Cells were preincubated with either cell culture medium alone or combined with X and/or XO. Next, the cells were washed with phosphate-buffered saline (room temperature, pH 7.4) and fixed in ethanol/methanol for 45 min and washed again. Subsequently, nonspecific binding sites were blocked by incubating the wells for 10 min with 1% goat serum (Sigma-Aldrich, Zwijndrecht, The Netherlands). Mouse monoclonal antibody to E-Selectin, intracellular adhesion molecule-1 (ICAM-1), or vascular cellular adhesion molecule-1 (VCAM-1) (ITK, Uithoorn, The Netherlands) in a dilution of 1 : 500 was added for 1 h, followed by the addition of biotinylated goat anti-mouse antibody (Sigma-Aldrich, Zwijndrecht, the Netherlands) in a dilution of 1 : 250. Increased sensitivity was obtained using the ExtrAvidin–Peroxidase system (Sigma-Aldrich, Zwijndrecht, the Netherlands). After washing away any free peroxidase, a substrate solution containing 2,2′-azino-bis(3-ethylbenzothiazoline-6-sulfonic acid) diammonium salt in 0.05 M citrate-phosphate buffer with urea hydrogen peroxide buffer with urea hydrogen peroxide was added. Incubation of endothelial cells without the primary antibody served as a negative control. As a positive control, the ExtrAvidin–Peroxidase system was added followed by substrate development without washing away the peroxidase. After 40 min the reaction was stopped with sodium fluoride and photometrical evaluation was performed with a computer-controlled ELISA reader at *λ*=405 nm.

### Apoptosis

To assess whether superoxide anions caused apoptosis in MEC a cell-death detection ELISA^plus^ kit (Roche Applied Science, Almere, The Netherlands) was used for the detection of cytoplasmic histone-associated DNA fragments. In short, endothelial cells were grown to confluence as described for the adhesion assays in 96-well flat-bottomed microtitre plates. The cells were preincubated with X and/or XO for 12 h and then lysated, whereafter 20 *μ*l of the lysate was transferred into Streptavidin-coated microplate wells. Eighty microlitre of immunoreagent containing biotinylated anti-histone and peroxidase-labelled anti-DNA antibodies was added into the wells followed by incubation on a plate shaker under gently shaking (300 r.p.m.) for 2 h at 15–25°C. Then the wells were washed thoroughly with incubation buffer and 100 *μ*l of 2,2′-azino-bis-(3-ethylbenzthiazoline-6-sulfonic acid) substrate was added. Plates were incubated for 15 min on a plate shaker at 250 r.p.m. where after photometric analysis at 405 nm was performed.

### Proliferation assay

To establish whether preincubation of MEC monolayers with superoxide anions was of influence on MEC cell number, the DNA content was determined using the bisbenzimide fluorescent dye (Roche Applied Science, Almere, The Netherlands) as previously described by Hofland *et al* (1990). Therefore, 2 × 10^4^ endothelial cells ml^−1^ were plated in 24-wells plates and after 1 day X–XO was added. At days 0, 1, 2, and 3 after the addition of X–XO wells were washed and plates were stored at −20°C until analysis.

### Statistical analysis

All data were evaluated using analysis of variance to determine overall differences between groups. The Dunnett post-test was carried out to compare between groups. *P*⩽0.05 was considered to be statistically significant. Experiments (*n*=6) were performed at least twice.

## RESULTS

### Evaluation of the model

**Figure 1:**
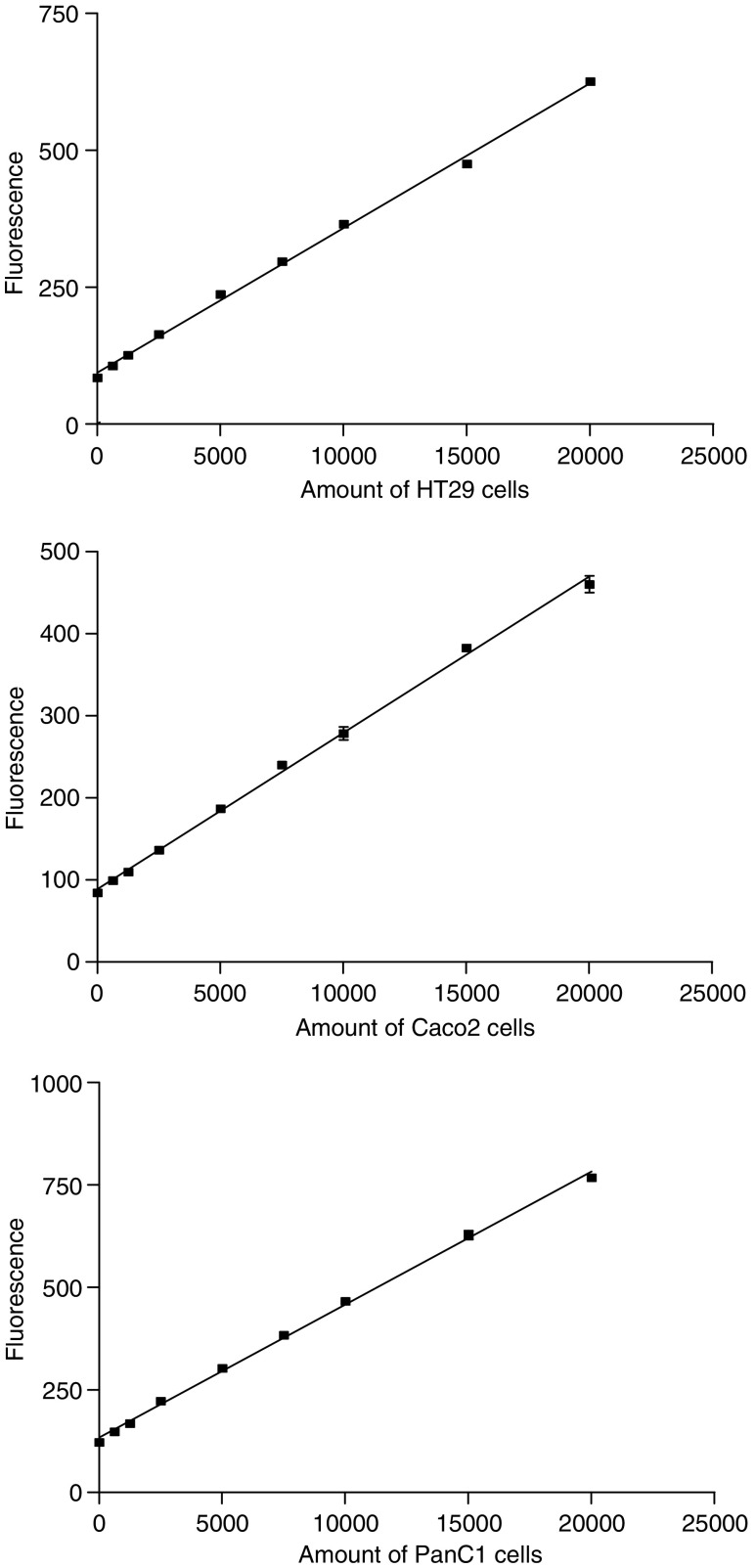
Calibration curves showing the fluorescence measurements (OD 485–530 nm) of calcein-AM-labelled tumour cells in order to quantify the amount of tumour cells. Data represent mean ±s.e.m. of duplicate wells.

Labelling tumour cells with calcein-AM did not decrease their viability (>95% using trypan blue). Dilution series of labelled tumour cells on endothelial monolayers showed a linear correlation (*r*^2^>0.99) between cell number and the level of fluorescence (Figure 1). Thus, by using such standard curves it became possible to estimate the number of adherent tumour cells in the experimental wells from the fluorescence intensity.

**Figure 2:**
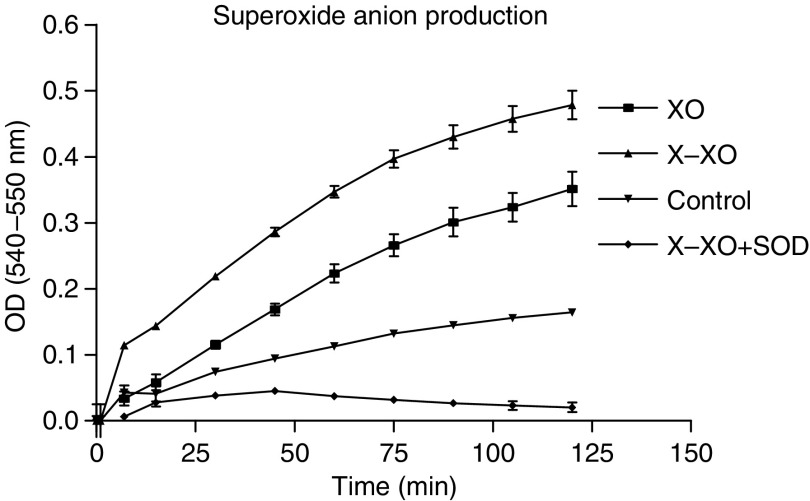
Validation of superoxide anion production by the ferricytochrome *c* reduction assay. Xanthine–xanthine oxidase was added to the wells with or without 400 U ml^−1^ SOD immediately followed by the addition of 75 *μ*M cytochrome *c*. Continuous measurements at 550–540 nm were carried out for 125 min. Data represent mean absorbance values (OD 540-550 nm) ±s.e.m. of quadriplate wells.

In our model, ferricytochrome *c* in the wells with X–XO was reduced at a rate of 0.32 nmol ml^−1^ min^−1^ as can be calculated from the results presented in Figure 2 using a molecular extinction coefficient of ferricytochrome *c* of 13.125 M^−1^ for a light path of 0.625 cm in the microtitre plate. The addition of SOD prevented the reduction of ferricytochrome *c* completely, indicating that the X–XO system indeed mainly generated superoxide and that 400 U ml^−1^ SOD is sufficient in this model to dismutate the formed superoxide anions. Interestingly, in the absence of X–XO still generated superoxide, but at a lower rate (Figure 2). In the assay with XO only in the absence of foetal calf serum, we found no superoxide production (data not shown), whereas in the presence of foetal calf serum, but without the addition of XO and extra X, MEC also were found to produce some superoxide (Figure 2). This made it likely that the foetal calf serum of the medium contained the necessary substrate X, and that MEC contain some endogenous XO (Figure 2).

### Adhesion to microvascular endothelial cells

Basal adhesion, that is, adhesion to non-preincubated MEC, was between 20 and 30% of added cells for HT29 and Caco2. For PanC1, basal adhesion was between 10 and 20%.

**Figure 3:**
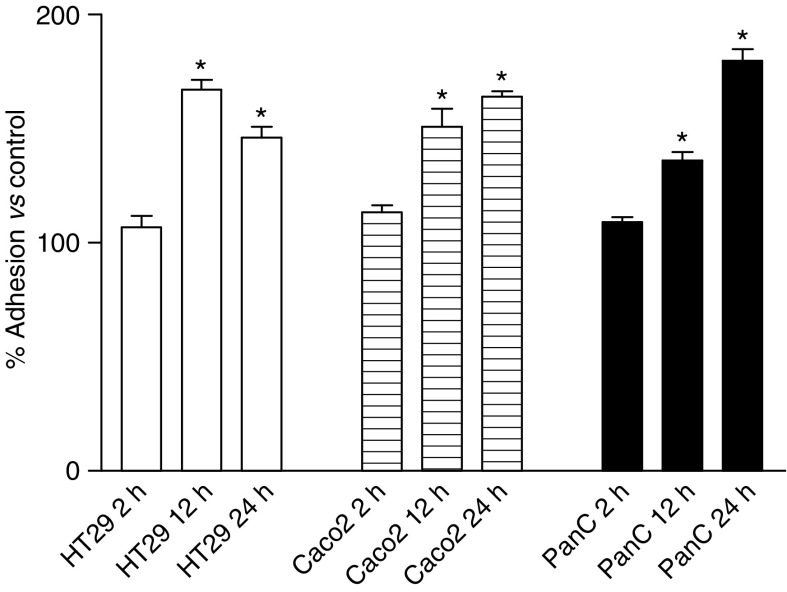
Tumour cell adhesion to MEC after preincubation of MEC with X–XO at varying time intervals. Means (*n*=6; % *vs* control) ±s.e.m. are shown. ^*^P<0.01 *vs* control.

Preincubation of MEC with the X–XO complex enhanced tumour cell adhesion (Figure 3). For PanC1, this enhancement occurred after 12 h preincubation and was increasing with longer preincubation times reaching a maximum of 180% *vs* control (untreated MEC) after 24 h of preincubation (*P*<0.01). Comparable results were found for Caco2 after X–XO preincubation of MEC with a tumour cell adhesion of 164% compared to basal adhesion (*P*<0.01). Maximal adhesion for HT29 occurred already after 12 h preincubation of MEC with X–XO and was 167% *vs* control (*P*<0.01).

**Figure 4:**
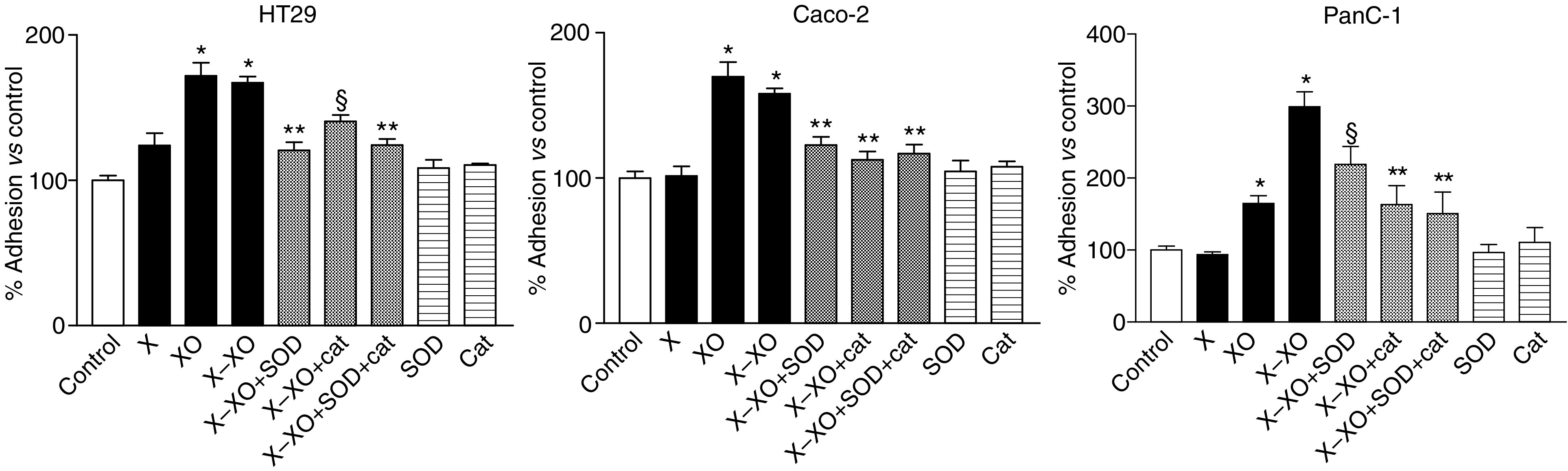
Adhesion of HT29 (**A**), Caco2 (**B**), and PanC1 (**C**) after 12 h preincubation of MEC with X, XO, and X–XO. The antioxidant enzymes SOD, catalase (cat) and the combination of both were added during the preincubation. Measurement of adhesion to an empty well (plastic) was determined as a negative control. Means (*n*=6; % *vs* control) ±s.e.m. are shown. ^*^P<0.01 *vs* control; ^**^P<0.01 *vs* X–XO; §*P*<0.05 *vs* X–XO.

Preincubation with X alone did not influence tumour cell adhesion for all three cell lines (Figure 4). However, preincubation with XO alone did enhance the adhesion of HT29 to 172%, of Caco2 to 170%, and of PanC1 to 128% *vs* control (all *P*<0.01) (Figure 4). This was not surprising, as XO in medium alone did produce superoxide anions (Figure 2), probably because foetal calf serum in the medium contains X, acting as a substrate for XO.

**Figure 5:**
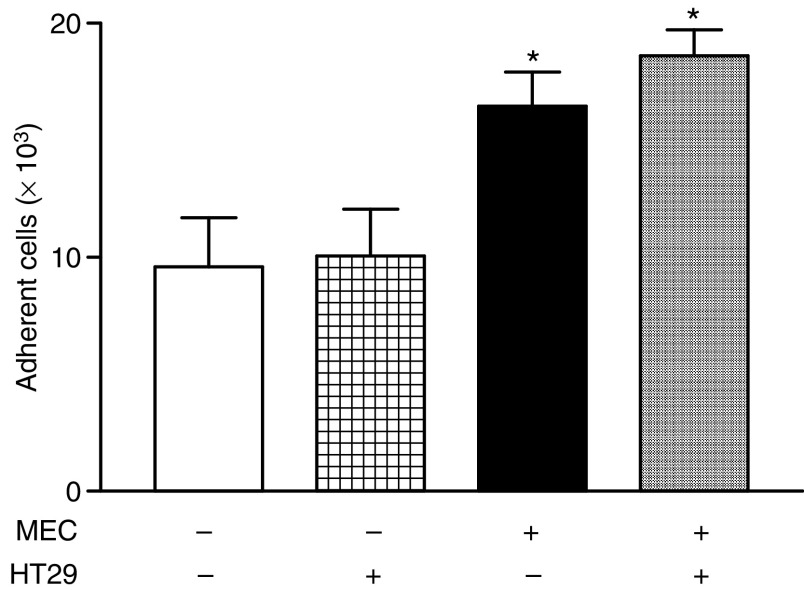
Adhesion of untreated HT29 *vs* HT29 preincubated with X–XO for 12 h (HT29+X–XO) to untreated endothelium (control) and endothelium preincubated with X–XO (X–XO). Means (*n*=6; % *vs* control) ±s.e.m. are shown. ^*^P<0.01 *vs* control.

Preincubation of HT29, Caco2, and PanC1 with the X–XO complex for 12 h did not enhance their adhesion to untreated or pretreated MEC statistically significantly (Figure 5; only data for HT29 are shown).

To verify if superoxide is the relevant ROS causing the enhanced adhesion we evaluated the effects of SOD in this model (Figure 4). Addition of SOD did decrease the enhanced adhesion of Caco2 to X–XO-treated MEC to nearly basal levels, from 158 to 116% (*P*<0.01). Comparable results were observed for HT29. SOD also decreased adhesion of PanC1 to X–XO-treated MEC, from 299 to 213% (*P*<0.05). As superoxide anions spontaneously dismutate into the stronger ROS hydrogen peroxide that may affect MEC on its turn, we studied the effect of catalase next. The results showed that catalase inhibited the enhanced tumour cell adhesion after X–XO preincubation effectively as well, that is, for HT29 adhesion decreased from 167 to 141% (*P*<0.05), for Caco2 from 158 to 113% (*P*<0.01), and for PanC1 from 299 to 163% (*P*<0.01). In combination both antioxidant enzymes did not gave an additional effect. The addition of SOD or catalase to untreated MEC did not decrease basal adhesion alone (Figure 4), indicating that the low level of superoxide production of MEC in culture medium was insufficient to act as an autocrine stimulus (Figure 2).

### Mechanism of adhesion

**Figure 6:**
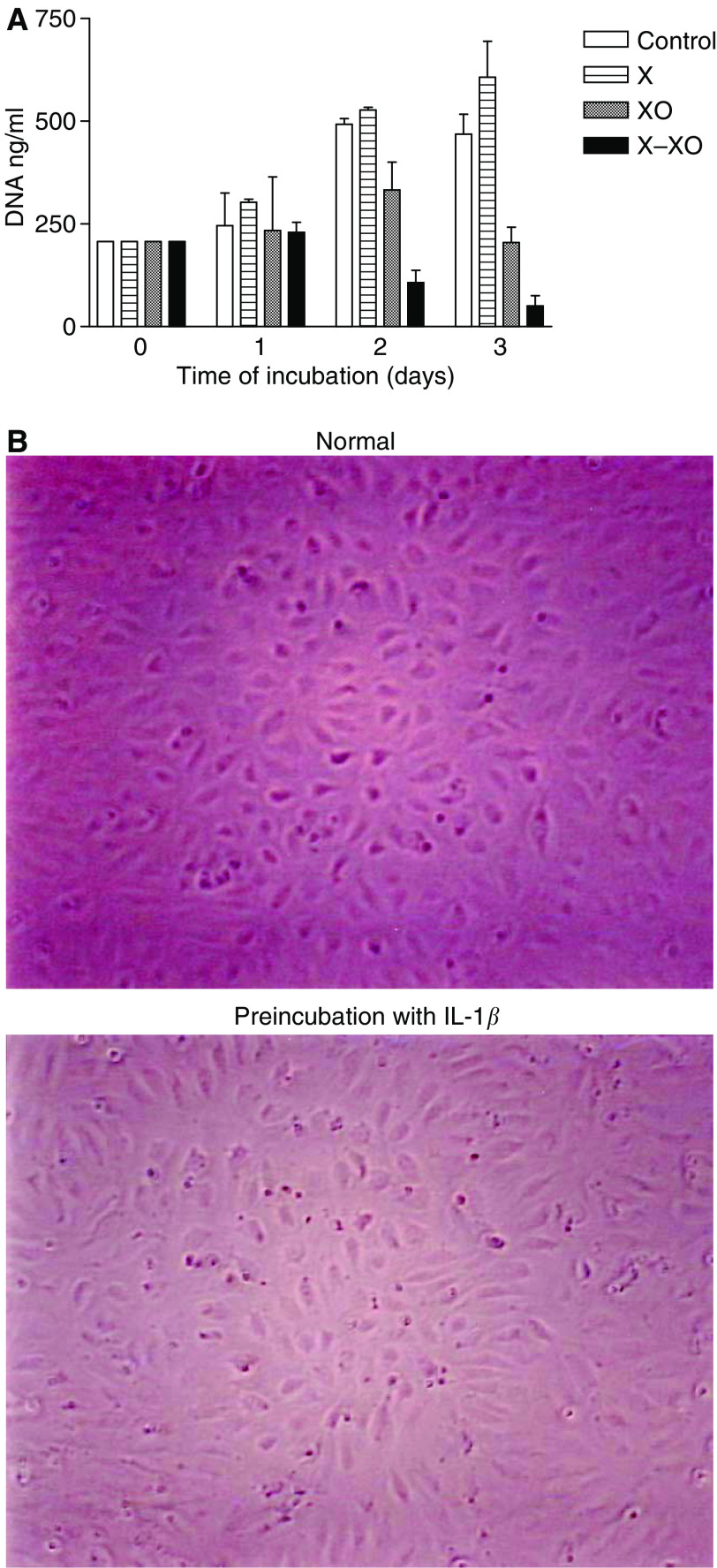
(**A**) MEC proliferation assay. DNA in ng ml^−1^ of MEC after 0, 1, 2, and 3 day(s) incubation with X, XO, and X–XO. Bars represent means (*n*=4). (**B**) Photographs of MEC monolayers: in medium (normal) and in medium preincubated with X–XO for 12 h. Comparable number of cells are seen.

**Figure 7:**
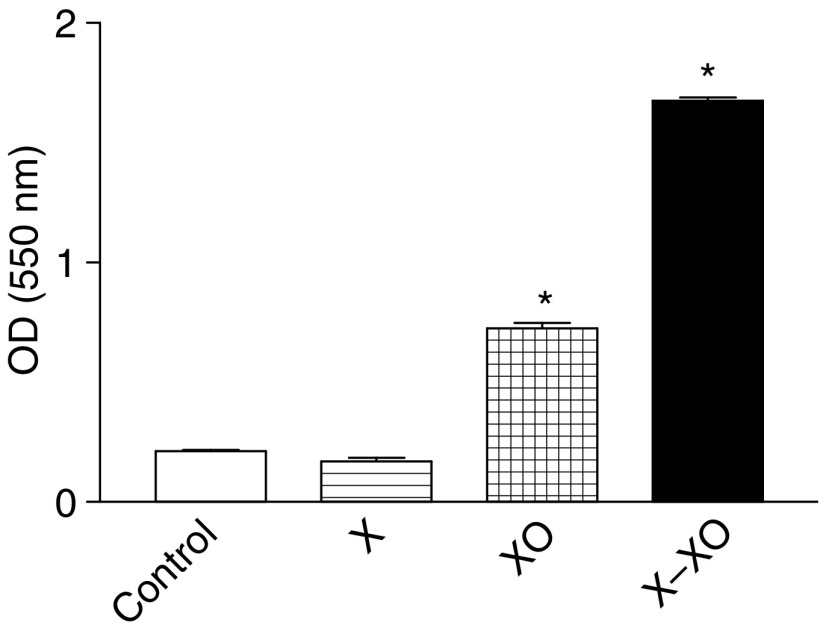
Measurement of apoptosis by ELISA in MEC after preincubation with X, XO, and X–XO for 12 h. Data represent mean absorbance values (OD 550 nm) ±s.d. of triplate wells. ^*^P<0.01 *vs* control.

To study if preincubation with X–XO influences the number of MEC we determined the course in the amount of DNA during an observation period of 3 days. The results showed a significant decline in the amount of DNA, not until 2 days of incubation with X–XO (Figure 6A). During the first 24 h of culture in presence or absence of X–XO however, the number of MEC as reflected by the amount of DNA did not change significantly. Photographs of the endothelial monolayers with or without preincubation with X–XO for 12 h also show that the number of endothelial cells is comparable (Figure 6B). On the other hand, preincubation with X–XO did lead to an increased apoptosis rate of 7.9 times the normal apoptosis rate (*P*<0.01), whereas XO only stimulated the apoptosis rate 3.7-fold (*P*<0.01) (Figure 7).

It was previously shown (Chirivi *et al*, 1994; Giavazzi, 1996; Orr *et al*, 2000; ten Kate *et al*, 2004) that the adhesion molecules E-Selectin, ICAM-1, and VCAM-1 on MEC and the ligands lymphocyte function-associated antigen-1, very late activation antigen-4 and CD44 on tumour cells play an important role in tumour cell adhesion to MEC and that the expression of these molecules can be induced by apoptosis (Hebert *et al*, 1998b; Chandra *et al*, 2003a).

**Figure 8:**
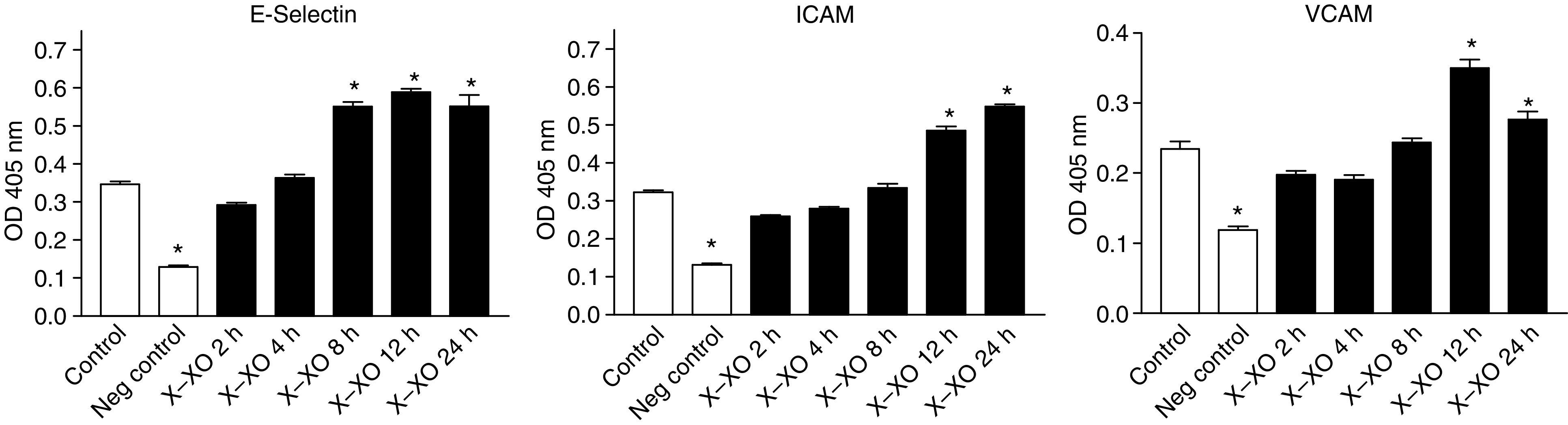
Adhesion molecule expression on MEC. After 2–24 h preincubation with X–XO, EIA with anti-E-Selectin, anti-ICAM,- and anti-VCAM-antibodies was performed. Bars represent the mean absorbance values (OD 405 nm) ±s.d. of quadriplate wells. ^*^P<0.01 *vs* control.

**Figure 9:**
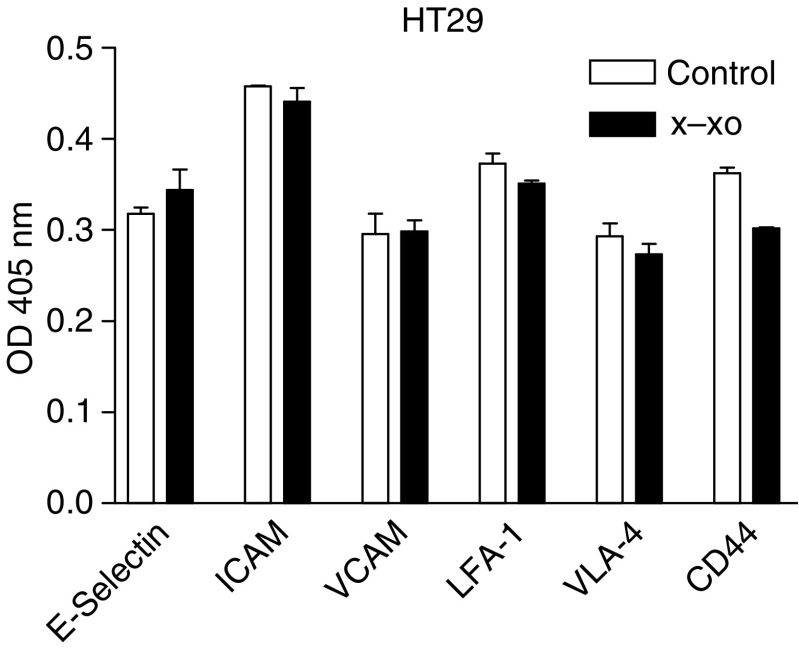
Adhesion molecule expression on HT29 colon carcinoma cells. After 12 h preincubation with X–XO, EIA was performed. Bars represent the mean absorbance values (OD 405 nm) ±s.d. of quadriplate wells.

In this model we found that nonstimulated MEC and tumour cells did express E-Selectin, ICAM-1, and VCAM-1 (Figures 8 and 9). After 8 h of preincubation with X–XO, enhanced E-Selectin expression on MEC was observed with a peak expression after 12 h of 1.66 times the expression on nonstimulated MEC (*P*<0.01). Increased ICAM-1 and VCAM-1 expression on MEC was observed later, namely after 12 h preincubation. Although ICAM-1 expression then increased still further to a maximum of 170% *vs* control (*P*<0.01), VCAM-1 expression declined after its peak expression of 149% *vs* control at 12 h of preincubation (Figure 6). None of the adhesion molecules under study showed enhanced expression on HT29 (Figure 9), Caco2, and PanC1 by X–XO preincubation (data not shown).

## DISCUSSION

ROS are known to play an important role in locoregional tumour recurrence after surgical trauma (van Rossen *et al*, 1999, 2000). In preliminary *in vivo* studies, we were able to detect a significant increase of ROS in peritoneal lavage fluid as well as in plasma after surgery, proving that indeed surgery induces not only a local enhancement of ROS, but also systemically (data not shown). So the inflammatory response after surgical trauma does not confine locally, but spreads out systemically and therefore it is interesting to investigate the role of ROS in the development of distant metastases after surgery.

Therefore, the X–XO complex was used to generate superoxide anions and in this way the influence of superoxide anions on tumour cell–endothelial cell interactions was studied. Exposure of microvascular endothelium to superoxide anions gave a substantial enhancement in tumour cell adhesion to the exposed endothelium comparable to the results found with PMN exposure, whereas exposure of tumour cells to superoxide anions had no effect on their adhesion to untreated endothelial cells.

We found that exposure of the MECs to superoxide anions led to an upregulation of the adhesion molecules E-Selectin, ICAM-1, and VCAM-1 on these cells. Enhancement of adhesion molecules on endothelial cells by exposure to ROS was also found by Bradley *et al* (1993) and Lo *et al* (1993). Both found significant increased ICAM-1 expression after preincubation with the ROS, although a relation with tumour adhesion was not investigated. Terada *et al* (1997) did not found an upregulation of ICAM-1 on the endothelium after a short preincubation period of 30 min with XO. This period of time is too short for completing synthesis of functional adhesion molecules, which is in accordance to our observation that a continuous exposure of the endothelium to ROS lasting minimally 12 h is necessary before endothelial cells show any increased expression of cellular adhesion molecules.

Exposure of the endothelium to superoxide anions resulted in a major increase of apoptosis. Apoptosis finally will result in cell death leading to loss of binding sites on the endothelium, but exposure of the underlying extracellular matrix as a substrate for binding sites for circulating tumour cells. However, it does not seem very likely that binding sites on the extracellular matrix contributed to the findings of the present study, because the preincubation of the endothelial cells lasted only 12 h during which the number of endothelial cells did not decrease.

The fact that endothelial cells undergoing apoptosis release interleukin -1 *β* (IL-1*β*) that via a paracrine loop in turn stimulates the expression of adhesion molecules on the endothelial cells (Hebert *et al*, 1998a; Chandra *et al*, 2003b) suggests that the following sequence of events for the recurrence of tumour cells at distant sites occur. Surgical trauma during the excision of a (primary) tumour leads to the activation of PMN. At distant sites these phagocytes by their massive production of ROS induce apoptosis of microvascular endothelium. Subsequently, by the (local) release of IL-1*β* the endothelial cells stimulate the expression of at least three major cellular adhesion molecules on their own cell membrane to which circulating tumour cells now easily can adhere and next form a metastasis.

The addition of SOD and/or catalase to ROS-exposed MEC decreased the enhanced tumour cell adhesion significantly. As both antioxidant enzymes decreased the adhesion to similar levels this means that not only superoxide anions, but also hydrogen peroxide are equally involved in this phenomenon. This indicates that in fact a third kind of ROS, namely the highly reactive hydroxyl radical, is the actual reactant. To generate hydroxyl radicals both superoxide and hydrogen peroxide are needed in the so-called transition metal catalysed Haber–Weiss reaction, and thus depleting one or the other ROS completely prevents the generation of the hydroxyl radical. Of note is that the addition of either SOD or catalase did not decrease the adhesion to basal levels. Incomplete scavenging of ROS by the antioxidant enzymes cannot account for that, because we showed here that adding SOD to the X–XO complex completely inhibited the generation of superoxide. Presumably the local increase in tension of molecular oxygen as a by-product of the inactivation of superoxide and hydrogen peroxide by SOD and catalase, or XO itself may have contributed to the incomplete reduction in the expression of the adhesion molecules.

In conclusion, the results of the present study suggest that ROS as a result of surgical trauma influence tumour recurrence at distant sites by increasing binding sites for tumour cells on the endothelium. This indicates that by tackling the production of ROS preventing tumour recurrence not only locally, but also at distant sites might be feasible.
